# Association Between Soil Patterns and Mortality with Distinct Types of Cancers and CVD Across the USA

**DOI:** 10.3390/life15060832

**Published:** 2025-05-22

**Authors:** Bingjie Qu, Qiaochu Xu, Linxi Yuan, Ying Chen

**Affiliations:** 1 Wisdom Lake Academy of Pharmacy, Xi’an Jiaotong-Liverpool University, Suzhou 215123, China; bingjie.qu20@student.xjtlu.edu.cn (B.Q.); qiaochu.xu21@student.xjtlu.edu.cn (Q.X.); 2 Institute of Systems, Molecular & Integrative Biology, University of Liverpool, Liverpool L69 7ZB, UK; 3 Department of Health and Environmental Sciences, Xi’an Jiaotong-Liverpool University, Suzhou 215123, China; linxi.yuan@xjtlu.edu.cn

**Keywords:** soil mineral, geochemistry, geochemical pattern, cause-specific death, cancer, cardiovascular condition, epidemiology

## Abstract

Mineral elements are essential for human health. Our previous study identified distinct clusters of health-related mineral elements in surface soil among different regions and demonstrated an association between these clusters and health profiles in the USA. The present study further explores the relationship between these mineral clusters and mortality from detailed specific types of cancers and cardiovascular diseases by using county-level data from 3080 counties across the USA. Utilizing multivariate regression models with adjustment for socio-demographic and geographical factors, our analysis of county-level data revealed that residents in the regions of ‘infertile’ cluster have higher mortality rates for most types of cancers (18/29) and cardiovascular conditions (4/10) compared with people who live elsewhere. Notably, this relationship is pronounced for several specific leading causes of death such as tracheal, bronchus, lung cancer (regression coefficient (99.5% CIs), 6.29 (4.46, 8.13)), prostate cancer (1.06 (0.53, 1.6)), cerebrovascular disease (3.15 (1.74, 4.55)), and hypertensive heart disease (1.23 (0.23, 2.23)). Our findings highlight the critical role of soil minerals in human health and underscore the need for integrating geochemical data in public health strategies and environmental management policies.

## 1. Introduction

Minerals (such as calcium (Ca), iron (Fe), manganese (Mn), selenium (Se), zinc (Zn), arsenic (As), cadmium (Cd), sodium (Na), etc.) play an essential role in human health and well-being, either positively or negatively [1,2]. Trace elements influence human health through several fundamental biological pathways. Essential minerals such as Se, Zn, Na, and Ca play critical roles in antioxidant defense, immune regulation, inflammation modulation, and cellular metabolism, which can impact cancer and cardiovascular disease development [3,4,5,6,7,8]. Conversely, toxic elements like As can contribute to disease pathogenesis through mechanisms involving oxidative stress, DNA damage, chronic inflammation, and impairment of cellular repair processes [9,10,11,12]. Thus, imbalances or deficiencies in these trace elements may underpin regional variations in cancer and cardiovascular disease incidence and mortality. The health of the local population has been shown to be affected by the presence of minerals in the environment [13,14].

Individual minerals associated with specific health outcomes have been extensively reviewed in the literature. However, a more systematic understanding of the spatial distribution of multiple health-related minerals over a large geographical area and their collective effects on health inequalities is lacking. In the previous work, we identified three geographic patterns of life expectancy-related minerals (As, Ca, Na, Se, and Zn) in stream sediment (or surface soil) samples across the contiguous USA, and found that, the cluster characterized by regions with the lowest concentrations of these five elements was associated with lower life expectancy at birth, higher mortality risk at all ages, and higher mortality rates from many specific causes, including the five leading causes of death [15]. To better understand the observed relationships and propose the possible mechanism, studies of specific types of disease are needed.

Cancer and cardiovascular disease represent a significant disease burden, with the highest mortality rates among all premature deaths in the USA [16,17,18,19,20,21]. In this extended analysis, we aimed to comprehensively assess the spatial associations between the geochemical clusters and cause-specific mortality rates for 29 different cancers and 10 different cardiovascular conditions. This study could provide additional knowledge for environmental epidemiology on surface soil mineral concentrations and human health.

## 2. Materials and Methods

### 2.1. Setting and Design

This ecological study primarily used two county-level databases to examine the association between soil trace element patterns and cause-specific mortality rates from specific types of cancers and cardiovascular diseases in 3080 USA counties (3080/3140, 98.1%). In addition, county-level data on demographic characteristics, socioeconomic factors, healthcare services, residential environmental conditions, and geographic location were collected and included in multivariate statistical analyses to adjust for potential confounding effects.

### 2.2. Database

The National Geochemical Survey (2008) provided county-level data from contiguous states on the concentrations of five soil minerals (As, Ca, Na, Se, Zn) relevant to life expectancy [15]. This nationwide project, conducted by the USA Geological Survey and its partners, used stream sediments and soils as primary samples, implemented consistent analytical methods, and aimed to analyze at least one sample from every 289 km^2^ at a depth of approximately 20 cm below the surface. Concentrations of As and Se were measured using hydride generation atomic absorption spectrometry (HG-AAS), while inductively coupled plasma-atomic emission spectrometry (ICP-AES) was used for the remaining minerals. All concentrations are reported in mg/kg [22].

In our previous study [15], we identified three geographical patterns of life expectancy-related minerals in stream sediment (or surface soil) samples across the contiguous USA. Specifically, we applied a multivariate linear regression model with backward selection to identify the soil minerals that were most associated with life expectancy at birth among a panel of elements. As a result, five minerals (As, Ca, Na, Se, and Zn) were identified for further exploration of the common patterns of mineral distribution across the USA using latent class analysis (LCA). It is important to note that they were not grouped based on biological function but rather due to their co-occurrence patterns in the real world. A three-cluster model was determined, with approximately 66.8% (n = 2056) of the counties falling into the ‘common’ cluster. Furthermore, about 24.0% (n = 739) of the counties fell into the ‘infertile’ cluster, while about 9.3% (n = 285) belonged to the ‘plentiful’ cluster. The cluster labels ‘common’, ‘infertile’, and ‘plentiful’ reflect relative mineral abundance in comparison to the national mean concentrations: ‘common’ corresponds to mineral levels close to the mean, ‘infertile’ denotes regions with significantly lower mineral concentrations, and ‘plentiful’ indicates areas with higher concentrations. The national distribution of these clusters is shown in Supplementary Figure S1.

Data of mortality rates for cancers and cardiovascular diseases were obtained from the Institute for Health Metrics and Evaluation which provides estimates of USA county-level cause-specific mortality rates from 29 distinct types of cancers (including lip and oral cavity cancer, nasopharynx cancer, other pharynx cancer, esophageal cancer, stomach cancer, colon and rectum cancer, liver cancer, gallbladder and biliary tract cancer, pancreatic cancer, larynx cancer, tracheal, bronchus and lung cancer, malignant skin melanoma, non-melanoma skin cancer, breast cancer, cervical cancer, uterine cancer, ovarian cancer, prostate cancer, testicular cancer, kidney cancer, bladder cancer, brain and nervous system cancer, thyroid cancer, mesothelioma, Hodgkin lymphoma, non-Hodgkin lymphoma, multiple myeloma, leukemia, and other neoplasms as a whole) and 10 different types of cardiovascular diseases (including rheumatic heart disease, ischemic heart disease, cerebrovascular disease, hypertensive heart disease, cardiomyopathy and myocarditis, atrial fibrillation and flutter, aortic aneurysm, peripheral vascular disease, endocarditis, and other types as a whole) [23]. The most recent data available for cause-specific mortality is from 2014.

USA national official sources provided data on county-level population characteristics, including variables, such as size, gender, age, and ethnicity. Socio-economic indicators such as educational attainment, median household income, unemployment and poverty rates, and gross domestic product per capita were collected. Healthcare services, including medical insurance coverage and physician-to-resident ratio, were included. The rural–urban continuum code was used to describe the residential environment, while geographical location was based on the latitude and longitude coordinates of the centroid of each county [24]. The data used were mainly from 2014 or closely related years.

### 2.3. Statistical Analysis

The geochemistry, cause-specific mortality and other data collected were mapped by county identification [15]. Descriptive and simple statistics (e.g., ANOVA and Chi-squared tests) of the studied variables, stratified by LCA-derived clusters, were first presented. Multivariate linear regression analyses were then performed to investigate the association between LCA-derived geochemical clusters (exposure variable) and cause-specific mortality rate for each type of cancer and cardiovascular disease (outcome variable). These analyses were adjusted for population demographics, socioeconomic factors, health services index, and residential and geographical environment to account for potential confounding effects. We confirm that all variables included in the present analysis were complete for the 3080 counties analyzed. The few counties (60/3140, 1.9%) with missing data in the geochemical or health datasets were excluded using a complete-case analysis approach.

All statistical analyses were performed using STATA (version 17). Result visualization was carried out using GraphPad Prism (version 9). To account for the multiple testing issue, *p* < 0.005 (instead of the conventional threshold, 0.05) was set as the statistical significance level for a conservative evaluation of associations.

## 3. Results

Concentration characteristics of Ca, Na, Zn, Se, and As, cause-specific mortality rates for 29 cancers and 10 cardiovascular diseases, and sociodemographic variables stratified by LCA-derived clusters of USA counties are shown in Table 1. Univariate analyses showed that the ‘infertile’ cluster, characterized by the lowest concentrations of the five minerals, had the highest mortality rates for most cancers (21/29) and cardiovascular conditions (8/10) compared with the other two clusters (Table 1).

**Figure 1:**
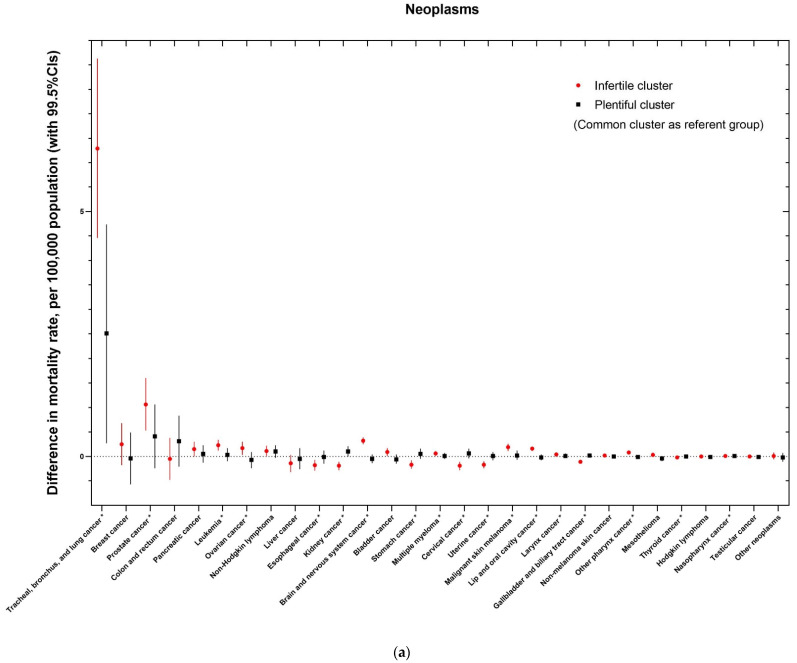

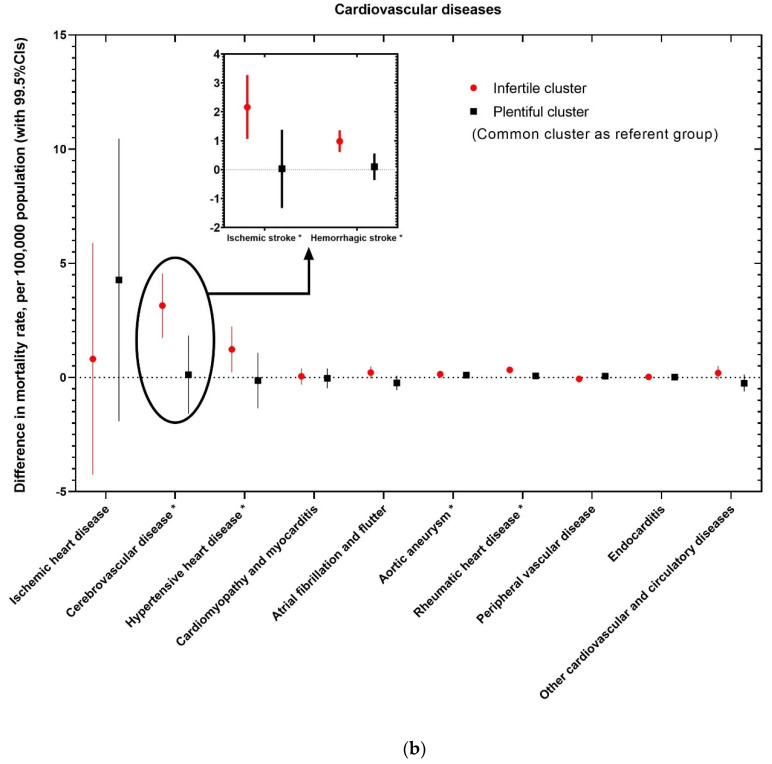
The associations between geochemical clusters with distinct types of neoplasms (**a**) and cardiovascular conditions (**b**) after adjustments for potential confounding factors. *, association reached the following statistical significance threshold, *p* < 0.005.

After adjustment for study covariates, with the ‘common’ cluster as the reference group, the results of multivariate linear regression analysis are shown in Figure 1. The adjusted association between the ‘infertile’ cluster and mortality rates for different cancer types (18/29) was demonstrated (Figure 1a). For example, the ‘infertile’ cluster showed significant positive associations with mortality rates for common cancers such as tracheal, bronchus and lung cancer (regression coefficient (99.5% confidence interval), 6.29 (4.46, 8.13)), prostate cancer (1.06 (0.53, 1.6)), and leukemia (0.23 (0.12, 0.34)).

Adjusted associations between the ‘infertile’ cluster and mortality rates for cardiovascular conditions were also presented (Figure 1b). Specifically, the ‘infertile’ cluster was significantly associated with increased mortality risk for cerebrovascular disease (3.15 (1.74, 4.55)), hypertensive heart disease (1.23 (0.23, 2.23)), aortic aneurysm (0.15 (0.08, 0.22)) and rheumatic heart disease (0.33 (0.23, 0.43)).

## 4. Discussion

The associations between individual minerals and health outcomes have been well-documented in the literature, yet a deeper, more comprehensive understanding of the spatial distribution of multiple minerals and their cumulative effects on health disparities is still emerging [13,15]. This gap highlights the importance of examining these elements in concert, as humans are typically exposed to combinations of minerals, rather than isolated exposures. Understanding these patterns across broad geographical areas is essential for creating a more accurate picture of health risks in real-world conditions. Epidemiological research has traditionally focused on single minerals and diseases due to challenges in designing, funding, and managing studies that include a comprehensive list of minerals and health indicators [25]. Using recently available high-quality open-access databases [22,26,27], our ecological study at the national scale in the USA has made an effort to evaluate the associations between multiple minerals and human health, providing novel insights into health disparities and potential directions for future public health strategies.

Our previous research showed that the specific geographic pattern—characterized by regions with the lowest concentrations of five life expectancy-related minerals (As, Ca, Na, Se, and Zn) in stream sediment (or surface soil)—was associated with higher overall mortality rates from cancer and cardiovascular disease, suggesting that soil mineral deficiencies may be a very important group of factors in health inequalities across the USA [15]. Our current findings extend this line of investigation by demonstrating a significant association between specific geochemical soil profiles and differential mortality rates from specific types of cancers and cardiovascular diseases. Notably, these associations are particularly pronounced for several leading causes of death, including tracheal, bronchus and lung cancer [28], prostate cancer [29,30], cerebrovascular disease [31], and hypertensive heart disease [28,32].

Our findings demonstrate significant associations between identified soil mineral clusters and mortality rates from specific cancers and cardiovascular diseases, yet they do not directly address the underlying biological mechanisms. Nevertheless, several plausible biological pathways could explain these associations. For instance, Se is a well-known antioxidant essential for human health, playing a crucial role in protecting cells from oxidative damage, modulating immune responses, and inhibiting carcinogenesis through its involvement in antioxidant enzyme systems such as glutathione peroxidase [33,34,35]. Deficiency in Se has been linked to increased risks for various cancers, including lung [36], prostate [4,34,37,38], cervical [39,40], thyroid [41,42], and colorectal cancers [37,43]. Additionally, Se deficiency has been linked to increased oxidative stress, endothelial dysfunction, and impaired cardiovascular health [44,45]. Epidemiological studies have found inverse associations between serum Se levels and stroke incidence, further supporting selenium’s cardiovascular protective role [46].

Zn is another essential mineral involved in numerous biological functions including antioxidant defense, immune function, DNA repair, and anti-inflammatory processes [5,47]. Deficiency in Zn has been associated with increased risks for several cancers, notably prostate cancer [48,49], and observational studies suggest that lower circulating Zn concentrations may be linked to a higher risk of lung cancer [50,51,52]. Moreover, Zn deficiency has also been implicated in adverse cardiovascular outcomes, such as increased stroke risk, potentially due to heightened oxidative stress and impaired vascular health [53]. Similarly, Ca contributes critically to cardiovascular health by regulating vascular contraction, blood pressure, and cardiac muscle function; inadequate Ca levels may exacerbate hypertension and cardiovascular disease risk [54,55].

The ‘infertile’ cluster is characterized by lower concentrations of beneficial minerals such as selenium and zinc, potentially contributing to higher observed mortality rates of specific cancers and CVDs through these biological pathways. It is important to clarify, however, that the inclusion of As within the same cluster primarily reflects statistical co-occurrence patterns across counties rather than similar biological functions. Arsenic is widely documented as a harmful environmental contaminant, known to promote carcinogenesis through oxidative stress, DNA damage, and epigenetic alterations [9,10,11,12]. Although As is grouped within the ‘infertile’ cluster due to statistical clustering of mineral concentrations, its relatively lower concentration in these regions suggests that the higher disease mortality rates observed are unlikely to be driven by As exposure. Instead, these associations likely reflect deficiencies in essential minerals such as selenium and zinc, which are critical for maintaining optimal health.

Minerals in surface soil may pose health impacts on residents through a soil–plant–human system due to their impact on agricultural productivity and the nutritional quality of locally grown food [56,57,58]. Residents were primarily exposed to soil minerals through consumption of local crops and livestock that absorb these minerals from soils [59,60,61]; thus, nutrient-poor soils can lead to deficiencies in dietary intake, increasing the population’s susceptibility to diseases related to malnutrition or impaired immune function [62]. Additionally, certain minerals can become airborne through dust inhalation or enter water supplies, providing secondary exposure pathways that may further influence health outcomes [63,64,65,66]. Taken together with our previous work [15], our research suggests that surface soil mineral content may contribute to inequalities in human populations across geographic regions, consistent with the growing recognition of the importance of environmental factors in determining health outcomes [67,68]. These findings provide valuable insights for further investigation into environmental epidemiology and beyond.

Our study contributes to the growing body of literature on environmental determinants of health by systematically evaluating geochemical patterns rather than individual mineral effects. However, it is important to acknowledge that, although we adjusted for several covariates, including socio-demographic, economic, healthcare, and geographical factors in our analysis, residual confounding from unmeasured or unavailable variables remains possible. Our research is an ecological study, providing initial and exploratory epidemiological evidence, but it is subject to ecological fallacy, where associations observed at the population level may not represent the relationships at the individual level. Nonetheless, our study design is made practical and economically feasible by using high-quality, publicly available databases across the contiguous United States. Future research using individual-level exposure assessments, longitudinal designs, and basic biological studies is necessary to strengthen the epidemiological evidence and to explore potential mechanistic pathways underlying the observed associations.

In summary, our findings highlight the critical role of soil geochemistry in regional health disparities. Compared to our previous study, which focused on geographic patterns of mineral deficiencies and their association with overall mortality rates from cancer and cardiovascular diseases, the current study takes a deeper, more targeted approach. While the earlier research identified general trends, the present study goes further by examining the associations between soil geochemical profiles and differential mortality rates from distinct types of cancer and cardiovascular diseases. By focusing on diseases such as tracheal, bronchus, and lung cancer, prostate cancer, cerebrovascular disease, and hypertensive heart disease, our current analysis offers more precise insights into specific mineral deficiencies and these health outcomes. Environmental surveillance in regions identified with mineral deficiencies may help mitigate geochemically linked health disparities and guide resource allocation.

## Figures and Tables

**Table 1:** Descriptive statistics of mineral concentrations, health measurements, and socio-demographics at the county level of USA by geographical pattern of minerals.

Characteristics		Number (%), or Mean (Standard Deviation)	Number (%), or Mean (Standard Deviation)	Number (%), or Mean (Standard Deviation)	Number (%), or Mean (Standard Deviation)	*p*-Value ^1^
		Total n = 3080	The “Common” Cluster n = 2056	The “Infertile” Cluster n = 739	The “Plentiful” Cluster n = 285	
Mineral concentration (mg/kg)						
Arsenic (As)		6.61 (5.45)	6.91 (3.24)	3.30 (1.72)	13.0 (12.99)	<0.001
Calcium (Ca)		17,167.71 (20,641.96)	17,787.26 (12,492.62)	2669.94 (2435.21)	50,290.75 (42,709.36)	<0.001
Selenium (Se)		0.34 (0.26)	0.34 (0.17)	0.20 (0.07)	0.68 (0.57)	<0.001
Sodium (Na)		6318.26 (4680.04)	7972.08 (4403.47)	1716.39 (1564.57)	6320.13 (3985.59)	<0.001
Zinc (Zn)		59.64 (48.18)	63.15 (22.57)	28.24 (14.55)	115.75 (122.96)	<0.001
Cause-specific mortality rate, number of deaths/100,000 population, 2014, (frequent ranking) ^2^						
Neoplasms						
Tracheal, bronchus, and lung cancer	(1)	62.58 (18.08)	59.12 (16.69)	73.81 (17.03)	58.48 (18.55)	<0.001
Breast cancer (females only)	(2)	26.31 (4.02)	25.53 (3.71)	28.89 (4.02)	25.25 (3.30)	<0.001
Prostate cancer (males only)	(3)	26.23 (4.98)	25.47 (4.13)	28.83 (6.43)	24.99 (3.72)	<0.001
Colon and rectum cancer	(4)	24.74 (4.29)	24.16 (4.06)	26.80 (4.33)	23.53 (4.09)	<0.001
Pancreatic cancer	(5)	12.86 (1.38)	12.66 (1.28)	13.58 (1.43)	12.49 (1.33)	<0.001
Leukemia	(6)	9.55 (0.9)	9.53 (0.89)	9.66 (0.83)	9.36 (1.10)	<0.001
Acute lymphoid leukemia	-	0.71 (0.12)	0.69 (0.12)	0.76 (0.11)	0.69 (0.12)	<0.001
Chronic lymphoid leukemia	-	2.91 (0.37)	2.93 (0.37)	2.88 (0.31)	2.84 (0.44)	<0.001
Acute myeloid leukemia	-	5.32 (0.51)	5.30 (0.50)	5.37 (0.46)	5.22 (0.63)	<0.001
Chronic myeloid leukemia	-	0.61 (0.06)	0.60 (0.05)	0.64 (0.05)	0.59 (0.06)	<0.001
Ovarian cancer (females only)	(7)	8.63 (0.94)	8.62 (0.92)	8.70 (0.99)	8.44 (0.91)	<0.001
Non-Hodgkin lymphoma	(8)	8.62 (0.94)	8.65 (0.93)	8.53 (0.85)	8.57 (1.09)	0.006
Liver cancer	(9)	6.36 (1.67)	6.07 (1.66)	7.21 (1.41)	6.20 (1.61)	<0.001
Esophageal cancer	(10)	5.49 (0.94)	5.55 (0.93)	5.43 (0.97)	5.28 (0.94)	<0.001
Kidney cancer	(11)	5.20 (0.76)	5.17 (0.75)	5.28 (0.71)	5.17 (0.87)	0.004
Brain and nervous system cancer	(12)	5.15 (0.61)	5.14 (0.58)	5.21 (0.69)	4.99 (0.58)	<0.001
Bladder cancer	(13)	5.10 (0.68)	5.12 (0.70)	5.05 (0.57)	4.99 (0.76)	0.001
Stomach cancer	(14)	4.43 (1.07)	4.21 (1.01)	5.09 (0.98)	4.27 (0.97)	<0.001
Multiple myeloma	(15)	3.99 (0.48)	3.92 (0.40)	4.24 (0.36)	3.84 (0.40)	<0.001
Cervical cancer (females only)	(16)	3.83 (1.08)	3.61 (0.99)	4.50 (1.05)	3.60 (0.96)	<0.001
Uterine cancer (females only)	(17)	3.74 (0.65)	3.77 (0.59)	3.63 (0.76)	3.67 (0.64)	<0.001
Malignant skin melanoma	(18)	3.39 (0.63)	3.38 (0.62)	3.40 (0.65)	3.40 (0.69)	0.703
Lip and oral cavity cancer	(19)	1.96 (0.48)	1.85 (0.39)	2.35 (0.51)	1.78 (0.36)	<0.001
Larynx cancer	(20)	1.39 (0.4)	1.30 (0.36)	1.68 (0.40)	1.26 (0.35)	<0.001
Gallbladder and biliary tract cancer	(21)	1.15 (0.23)	1.17 (0.24)	1.08 (0.14)	1.17 (0.24)	<0.001
Non-melanoma skin cancer	(22)	1.08 (0.22)	1.05 (0.21)	1.14 (0.22)	1.07 (0.23)	<0.001
Other pharynx cancer	(23)	1.02 (0.27)	0.95 (0.22)	1.24 (0.30)	0.91 (0.20)	<0.001
Mesothelioma	(24)	0.98 (0.35)	1.01 (0.34)	0.88 (0.39)	0.95 (0.28)	<0.001
Thyroid cancer	(25)	0.55 (0.05)	0.55 (0.05)	0.53 (0.04)	0.55 (0.05)	<0.001
Hodgkin lymphoma	(26)	0.4 (0.05)	0.38 (0.04)	0.41 (0.05)	0.37 (0.04)	<0.001
Nasopharynx cancer	(27)	0.29 (0.10)	0.26 (0.09)	0.38 (0.10)	0.26 (0.09)	<0.001
Testicular cancer (males only)	(28)	0.28 (0.06)	0.28 (0.05)	0.28 (0.06)	0.26 (0.05)	<0.001
Other neoplasms	-	6.22 (0.64)	6.17 (0.59)	6.43 (0.67)	5.99 (0.70)	<0.001
Cardiovascular diseases						
Ischemic heart disease	(1)	173.81 (47.17)	166.80 (45.42)	196.54 (45.70)	165.33 (44.63)	<0.001
Cerebrovascular disease	(2)	53.37 (11.4)	51.47 (10.27)	59.80 (12.31)	50.34 (10.4)	<0.001
Ischemic stroke	-	36.24 (8.2)	35.31 (7.64)	39.44 (8.89)	34.60 (8.08)	<0.001
Hemorrhagic stroke	-	17.13 (4.06)	16.16 (3.36)	20.35 (4.46)	15.74 (3.01)	<0.001
Hypertensive heart disease	(3)	10.18 (7.85)	8.85 (6.35)	14.39 (10.55)	8.74 (5.14)	<0.001
Cardiomyopathy and myocarditis	(4)	7.92 (3.12)	7.36 (2.81)	9.71 (3.38)	7.21 (2.73)	<0.001
Atrial fibrillation and flutter	(5)	7.56 (1.85)	7.72 (1.81)	7.20 (1.38)	7.35 (2.74)	<0.001
Aortic aneurysm	(6)	4.33 (0.62)	4.35 (0.62)	4.27 (0.54)	4.25 (0.70)	0.002
Rheumatic heart disease	(7)	3.43 (0.83)	3.28 (0.65)	3.92 (1.09)	3.20 (0.67)	<0.001
Peripheral vascular disease	(8)	2.68 (0.69)	2.62 (0.71)	2.85 (0.59)	2.63 (0.65)	<0.001
Endocarditis	(9)	2.59 (0.59)	2.59 (0.60)	2.63 (0.51)	2.51 (0.61)	0.021
Other cardiovascular and circulatory diseases	-	12.44 (2.23)	12.43 (2.28)	12.72 (2.01)	11.74 (2.28)	<0.001
Socio-demographics						
Population characteristics						
Size, n		101,310 (324,395)	95,968 (320,031)	79,131 (211,914)	197,354 (521,857)	<0.001
Gender, male %		50.04 (2.22)	50.09 (1.96)	49.79 (2.68)	50.26 (2.59)	0.002
Ethnicity, white alone %		85.53 (15.78)	89.01 (13.15)	74.42 (18.42)	89.25 (11.99)	<0.001
Age, %						
0–9 years		12.22 (2.09)	12.22 (2.19)	12.18 (1.77)	12.31 (2.10)	0.642
10–19 years		12.89 (1.67)	12.91 (1.67)	12.78 (1.57)	13.01 (1.86)	0.090
20–29 years		12.31 (3.31)	12.01 (3.08)	12.68 (2.96)	12.13 (2.86)	<0.001
30–39 years		11.53 (1.64)	11.39 (1.58)	11.76 (1.61)	11.88 (1.95)	<0.001
40–49 years		12.2 (1.46)	12.0 (1.42)	12.63 (1.33)	12.49 (1.68)	<0.001
50–59 years		14.61 (1.65)	14.73 (1.69)	14.24 (1.43)	14.63 (1.74)	<0.001
60–69 years		12.28 (2.45)	12.38 (2.50)	12.05 (2.20)	12.08 (2.54)	0.003
70–79 years		7.44 (1.99)	7.49 (1.97)	7.41 (2.05)	7.05 (1.88)	0.002
≥80 years		4.53 (1.50)	4.75 (1.53)	3.98 (1.18)	4.30 (1.61)	<0.001
Socio-economics						
Educational level (age ≥ 25), %						
Less than a high school diploma		13.42 (6.32)	12.42 (6.26)	16.73 (5.51)	11.99 (5.61)	<0.001
A high school diploma only		34.32 (7.18)	34.0 (7.27)	35.72 (6.41)	32.98 (7.75)	<0.001
Completing a college or associate’s degree		30.72 (5.19)	31.38 (5.41)	28.92 (4.20)	30.55 (4.74)	<0.001
A bachelor’s degree or higher		21.54 (9.41)	22.19 (9.21)	18.62 (8.63)	24.46 (10.83)	<0.001
Median household income (annual), USD		46,963.46 (12,008.42)	48,207.46 (11,663.56)	41,724.81 (10,910.38)	51,572.92 (12,874.3)	<0.001
Unemployment rate, %		6.22 (2.22)	5.91 (2.26)	7.33 (1.87)	5.50 (1.67)	<0.001
Poverty rate, %		16.87 (6.43)	15.78 (5.94)	20.74 (6.51)	14.69 (5.42)	<0.001
Gross domestic product per capita (annual, USD)		48,919 (136,007)	52,529 (158,554)	34,368 (32,101)	60,605 (123,203)	<0.001
Healthcare service						
Medical insured population (age < 65), %		85.72 (5.04)	86.63 (5.04)	83.24 (3.96)	85.51 (5.38)	<0.001
Physicians (per 1000 population), n		1.21 (1.62)	1.23 (1.75)	1.03 (1.14)	1.48 (1.66)	0.001
Residential environment and location						
Rural–Urban Continuum Code						<0.001
1 (Metro areas, 1 million population or more)		428 (13.90)	242 (11.77)	102 (13.80)	84 (29.47)	
2 (Metro areas, 250 thousand to 1 million population)		375 (12.18)	248 (12.06)	104 (14.07)	23 (8.07)	
3 (Metro areas, population fewer than 250 thousand)		350 (11.36)	234 (11.38)	102 (13.8)	14 (4.91)	
4 (Urban population of 20 thousand or more, adjacent to a metro area)		213 (6.92)	152 (7.39)	43 (5.82)	18 (6.32)	
5 (Urban population of 20 thousand or more, not adjacent to a metro area)		88 (2.86)	61 (2.97)	17 (2.30)	10 (3.51)	
6 (Urban population of 2500 to 19,999, adjacent to a metro area)		587 (19.06)	362 (17.61)	174 (23.55)	51 (17.89)	
7 (Urban population of 2500 to 19,999, not adjacent to a metro area)		418 (13.57)	306 (14.88)	78 (10.55)	34 (11.93)	
8 (Completely rural or less than 2500 urban population, adjacent to a metro area)		217 (7.05)	145 (7.05)	57 (7.71)	15 (5.26)	
9 (Completely rural or less than 2500 urban population, not adjacent to a metro area)		404 (13.12)	306 (14.88)	62 (8.39)	36 (12.63)	
Latitude		38.3 (4.83)	39.79 (4.43)	34.01 (3.04)	38.75 (4.87)	<0.001
Longitude		−91.51 (11.41)	−93.16 (12.37)	−86.09 (5.95)	−93.77 (10.61)	<0.001

^1^ *p* values are from ANOVA or Chi-squared tests. ^2^ Ranking, based on the average mortality rates, ranked separately for cardiovascular diseases and neoplasms, starting from the most frequent cause of death.

## Data Availability

The datasets used during the current study are available from the corresponding author on reasonable request.

## Supplementary Materials

The following supporting information can be downloaded at: https://www.mdpi.com/article/10.3390/life15060832/s1, Table S1: The source of demographic and socioeconomic data. Figure S1: Geographical pattern of the studied mineral clusters across the USA.
