# Supplementary material for: Association Between Soil Patterns and Mortality with Distinct Types of Cancers and CVD Across the USA
Source: Life (Basel). 2025 May 22;15(6):832. doi: 10.3390/life15060832 (PMC12194629; doi:10.3390/life15060832)
Supplement: Supplementary file 1 [file life-15-00832-s001.zip › life-3526467-supplementary.pdf]

Supplementary Information

Supplementary Table 1. The source of demographic and socioeconomic data

Variables	Source	Data link
Population size	Population estimates for the U.S., States, and counties, 2010-19.	https://www.ers.usda.gov/data-products/county-level-data-sets/download-data.aspx
Gender, age and ethnic	US Census Bureau. Annual County Resident Population Estimates by Age, Sex, Race, and Hispanic Origin: April 1, 2010 to July 1, 2014.	https://www2.census.gov/programs-surveys/popest/datasets/2010-2014/counties/asrh/
Educational level	U.S. Census Bureau, 1970, 1980, 1990, 2000 Censuses of Population, and the 2014-18 American Community Survey 5-yr average county-level estimates.	https://www.ers.usda.gov/StaticErrorPages/custom404.htm?aspxerrorpath=/data-products/county-level-data-sets/download-data
Median household income	U.S. Census Bureau, Small Area Income and Poverty Estimates (SAIPE) Program.	https://www.census.gov/data/datasets/2020/demo/saipe/2020-state-and-county.html
Unemployment rate	U.S. Department of Labor, Bureau of Labor Statistics, Local Area Unemployment Statistics (LAUS).	https://www.bls.gov/lau/#tables
Poverty rate	U.S. Census Bureau, Small Area Income and Poverty Estimates (SAIPE) Program.	https://www.census.gov/data/datasets/time-series/demo/sahie/estimates-acs.html
Medical insured population	U.S. Census Bureau, 2008-2019 small area health insurance estimates (SAHIE) Program.	https://www.census.gov/data/datasets/time-series/demo/sahie/estimates-acs.html
Physicians	Health Resources and Services Administration. Area Health Resource Files: Total active non-federal MDs.	https://www.census.gov/data/datasets/time-series/demo/sahie/estimates-acs.html
Rural-Urban Continuum Code	USDA ERS - Rural-Urban Continuum Codes.	https://data.hrsa.gov/data/download

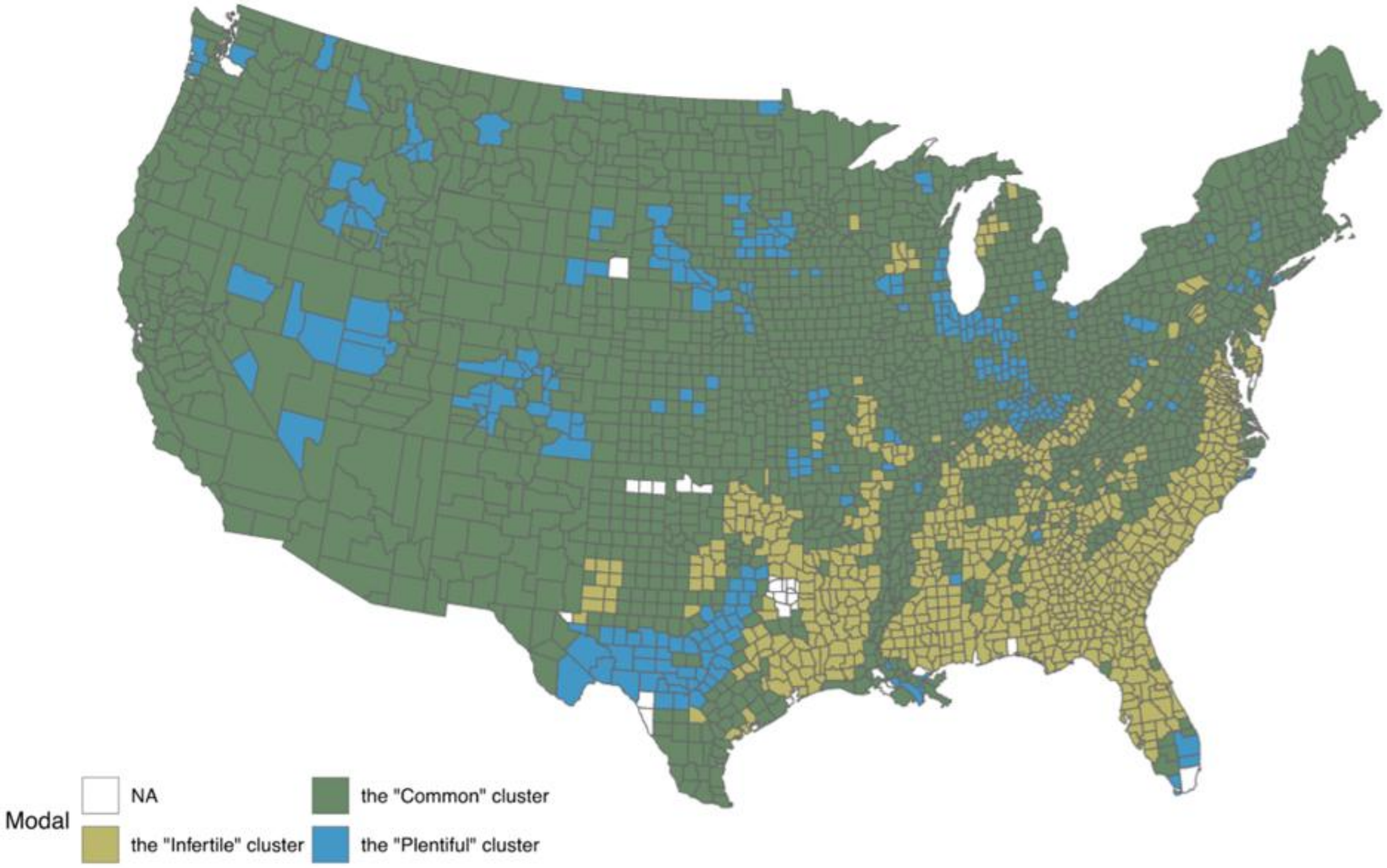

Supplementary Figure 1. Geographical pattern of the studied mineral clusters across the USA
